# Descriptive CBCT Findings of Maxillary Sinus Mucosal Changes in Patients Undergoing Sinus Floor Elevation: A Retrospective Observational Study

**DOI:** 10.3390/dj14060340

**Published:** 2026-06-02

**Authors:** Nicole Mckeever, Rabia Khan, Cemal Ucer, Simon Wright, Adam Spacey

**Affiliations:** 1 ICE Postgraduate Dental Institute and Hospital, 24 Furness Quay, Salford M50 3XZ, UK; nicole.mckeever@bupadentalcare.co.uk (N.M.); ucer@icedental.institute (C.U.); profwright@glencairndental.co.uk (S.W.); 2 School of Health and Society, University of Salford, Salford M5 4WT, UK; a.spacey@salford.ac.uk

**Keywords:** cone-beam computed tomography, dental implants, maxillary sinus, sinus floor elevation, mucosal thickness

## Abstract

**Objectives:** To assess the prevalence of maxillary sinus pathology in patients requiring sinus floor elevation to aid dental implant treatment. The study aimed to identify the degree, morphology, and location of mucosal thickening and its relation to ostium patency, as well as any odontogenic pathology contributing to mucosal thickening. **Methods:** This study was conducted at the ICE Postgraduate Dental Institute and Hospital, England, UK, between February and April 2025. cone-beam computed tomography (CBCT) scans of 20 patients who were partially dentate and edentulous who attended ICE for sinus floor elevation (SFE) between August 2023 and March 2025 were retrospectively examined. Mucosal thickening >2 mm was considered pathological. The CBCT scans of 26 maxillary sinuses were analysed for the following mucosal thickening characteristics: degree, morphology, and location of mucosal thickening, ostium patency, presence of odontogenic pathology, and need for onward referral to otorhinolaryngology. Data was also collected in relation to patient demographics and CBCT parameters. Descriptive statistics were used to summarise the data. **Results:** Of the 26 sinuses examined in the study, 69% presented with mucosal thickening greater than 2 mm. The incidence of mucosal thickening was higher in male patients. Polypoid mucosal thickening was the most common morphology observed in 46%, and circumferential thickening was the most common location observed in 50%. Ostium obstruction was seen in 12.5% of sinuses that had polypoid, circumferential thickening >10 mm. Other incidental findings included apical pathology, periodontal disease, dental implants breaching the sinus floor, and an antrolith. **Conclusions:** The study found a high incidence of mucosal thickening in patients undergoing sinus floor elevation. The degree and morphology of mucosal thickening, along with ostium patency and the presence of odontogenic pathology, are important factors to consider in preoperative assessment. Large field of view CBCT scans are required to allow visualisation of the osteomeatal complex. Collaboration between dental implantologists and otorhinolaryngologists is crucial for managing patients with sinus pathology and ensuring successful outcomes in SFE. Further larger prospective studies with clinical correlation are needed to better evaluate the association between sinus pathology, ostium patency, and sinus floor elevation outcomes.

## 1. Introduction

Dental implants are a well-established treatment option for the rehabilitation of partially or completely edentulous patients, with long-term survival rates comparable to tooth-supported fixed dental prostheses [1]. Successful implant placement requires sufficient bone volume; however, the posterior maxilla often presents challenges due to decreased alveolar height resulting from maxillary sinus pneumatization and alveolar bone atrophy following extractions [2]. Sinus augmentation procedures, such as lateral wall or transcrestal sinus floor elevation (SFE), are predictable methods for increasing bone height in this region [3].

Maxillary sinus anatomy and pathology are of particular clinical relevance in SFE procedures. Variations such as accessory ostia, as well as pathologies including mucosal thickening, mucous retention cysts, and complete sinus opacification, can increase the risk of intraoperative complications, postoperative morbidity, and implant failure [4,5,6,7]. Mucosal thickening is the most frequently observed finding on cone-beam computed tomography (CBCT), with reported prevalence ranging from 14.3% to 93.1% [4,8]. Odontogenic pathology accounts for approximately 25–40% of chronic maxillary sinus disease [4,8,9], further emphasising the importance of thorough preoperative evaluation.

CBCT is considered the preferred imaging modality for assessing sinus anatomy, residual alveolar bone, and pathology prior to SFE [10,11]. Compared to panoramic imaging, CBCT provides three-dimensional detail, enabling the detection of sinus septa, lateral wall thickness, posterior superior alveolar artery location, and odontogenic pathology, all of which can influence surgical planning [4,12,13,14]. However, there is no consensus regarding the optimal CBCT field of view (FOV) for pre-SFE assessment. While some advocate for a large FOV to include the osteomeatal complex for evaluating ostium patency [8,15,16], others caution against increased radiation exposure and the inclusion of anatomical regions outside the dentist’s diagnostic expertise [17,18].

The threshold for defining pathological mucosal thickening also varies across studies, with cut-offs reported between >1 mm and >3 mm [4,8], although >2 mm remains the most widely adopted criterion [19]. Thickening >4 mm has been associated with an increased risk of membrane perforation during SFE [20]. Morphological variations in thickening, such as polypoid, circumferential, or irregular forms, are linked to a higher likelihood of ostium obstruction [15,16]. Additional findings, such as calcifications [21,22] and odontogenic sinusitis, can further complicate surgical outcomes if unrecognised preoperatively [9,23,24]. In addition, assessment of maxillary sinus ostium patency is a critical factor in evaluating the risk of postoperative complications, as impaired drainage may predispose to sinusitis following sinus floor elevation. However, accurate assessment of the osteomeatal complex requires an adequate field of view, and CBCT scans with a limited (small) FoV may not allow reliable visualisation of the ostium, thereby restricting comprehensive preoperative evaluation.

Referral to ear, nose, and throat (ENT) specialists is recommended in specific scenarios, including cases of thickened or polypoid mucosa with ostium obstruction, irregular or circumferential thickening >5 mm, air–fluid levels, or suspected mucocele formation [15,16,17]. The framework proposed by Janner et al. (2020) [18] provides structured guidance for referral, though its application in dental implant contexts remains under-investigated.

Given the high reported incidence of sinus and odontogenic pathology on CBCT, their potential influence on SFE outcomes, and the variability in diagnostic and referral protocols, further evidence is needed to guide clinical decision-making. This retrospective descriptive study aims to evaluate the prevalence and characteristics of maxillary sinus and odontogenic pathology in CBCT scans of patients requiring SFE to aid dental implant treatment. Specifically, it seeks to assess the degree, morphology, and location of mucosal thickening; identify associated odontogenic pathology; determine ostium patency; evaluate CBCT FOV adequacy; and apply the ENT referral framework of Janner et al. (2020) [18] to the study population. The findings aim to enhance preoperative planning, reduce complications, and contribute to the evidence base supporting safer, more predictable outcomes in SFE procedures.

## 2. Materials and Methods

### 2.1. Study Design and Setting

This retrospective descriptive study was conducted at ICE Postgraduate Dental Institute and Hospital, England, UK, between February and April 2025. Following application for ethical approval from the Ethics Administration at the University of Salford in November 2024, ethical approval was granted by the University of Salford ethics committee on 3 February 2025.

### 2.2. Patient Selection

All patients who attended ICE PostgraduateDental Institute and Hospital, England, UK, between August 2023 and March 2025 for SFE to aid dental implant treatment in the posterior maxilla were consecutively enrolled. This study included a preselected cohort of patients referred for CBCT imaging in the context of implant planning and sinus floor elevation, representing a higher-risk group rather than a general population sample.

Inclusion criteria are as follows.

- Patients who had CBCT scans taken of unilateral or bilateral maxillary sinuses prior to SFE to aid dental implant treatment;
- Male and female patients;
- Patients aged over 18 years old;
- Patients who were partially dentate or edentulous.

Exclusion criteria are as follows.

- Low-resolution images, where pathologies or anatomy of the maxillary sinus could not be clearly identified, and
- those with imaging artefacts that impaired visualisation of the sinuses, such as those created by movement or metallic objects, e.g., fillings or crowns.

### 2.3. Data Collection

#### 2.3.1. Cone-Beam Computed Tomography

CBCT images were obtained using an Accuitomo (J.Morita MFG CORP, Kyoto, Japan) CBCT scanner under specified exposure conditions. The field of view (FoV) ranged from 4 × 4 × 4 cm to 8 × 8 × 8.6 cm, depending on the clinical indication, with a voxel size ranging from 0.08 mm to 0.2 mm.

Image analysis was performed using quantitative radiology software i-Dixel (version 2.0, J. Morita, Kyoto, Japan; 2026), which allows multiplanar reconstruction (MPR) in axial, coronal, and sagittal planes so that the sinus could be viewed in three dimensions. 

CBCT Parameters

Twenty-six sinuses in total were examined; six patients had CBCT of bilateral sinuses, 14 had a CBCT of a single sinus, resulting in 16 left and ten right maxillary sinuses being included. All patients were partially dentate except two who were edentulous. Three of the patients who were partially dentate also had dental implant/s in situ. The area captured by the CBCT scans, also known as the field of view (FOV), ranged from small 4 × 4 × 4 to medium 8 × 8 × 8.6 (Table 1). Of the patients examined, eight of the CBCT scan FOVs did not extend to enable visualisation of the ostium or the osteomeatal complex. Seven of the CBCT scans enabled viewing of the complete osteomeatal complex, whilst five allowed partial visualisation of the complex, which included the ostium. The scans that allowed complete or partial visualisation of the osteomeatal complex had a FoV of 8 × 8 × 8.6 cm or 8 × 8 × 8.5 cm. However, in four cases, despite using an 8 × 8 × 8.5 cm FoV, the ostium and osteomeatal complex could not be reliably visualised. This did not exclude these scans from the study, as the primary aim was to assess mucosal thickening and associated radiographic features; however, it limited the assessment of ostium patency in these cases and represents a methodological limitation rather than a failure to meet inclusion criteria. Of the 12 sinuses that had >5 mm mucosal thickening, four did not have an adequate field of view to enable visualisation of the ostium. The tube voltage for all scans was set at 90 kilovolts (kV), the exposure times ranged from 9.3 to 9.4 s, the tube current ranged between 7 and 8 milliAmps (mA), and the absorbed dose was between 3.69 and 9.84 milliGray (mGy).

The CBCT scans were examined at ICE by R.K., who had completed level 1 (core training in dental CBCT) and level 2 (further training for operators performing dental CBCT imaging and further training in dental CBCT justification and image interpretation), as advised by the Faculty of the General Dental Practitioner (FGDP) and Public Health England (PHE) in their guidance notes for dental practitioners on the safe use of X-ray equipment (PHE and FGDP (UK), 2020). The investigator was not involved in the treatment or follow-up of the patients included in the study. All data were independently verified by R.K to ensure accuracy and objectivity. Data collection during image analysis included demographic information of patients, including age and sex, as well as the degree, morphology, and location of any mucosal thickening, and the presence of any apical pathology or periodontal disease. Each sinus was assessed on an individual basis, and the status of the dentition and whether the ostium was observable and patent on the CBCT scan was also recorded, along with the exposure parameters of the CBCT scans taken. All the data was collected into a Microsoft Excel spreadsheet.

#### 2.3.2. Mucosal Thickening

**Figure 1:**
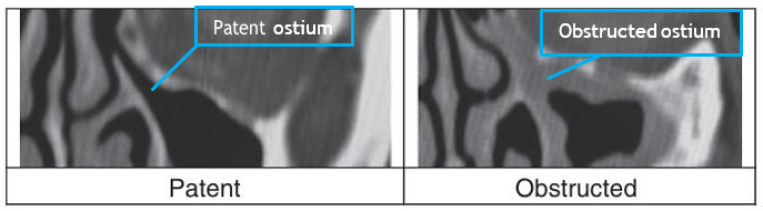
Coronal section of CBCT scans depicting the difference in appearance between a patent and obstructed ostium, adapted from Carmeli et al. [16].

In relation to mucosal thickening, any thickening exceeding 2 mm was classified as pathological, and, as per the work by Shanbhag et al. [17], was graded into 4 categories of metric thickening: <2, 2.1–5, 5.1–10, or >10 mm. This grading system was chosen as opposed to the method chosen by Carmeli et al. (2011) [16], in which thickening was graded <5 mm, <10 mm, <15 mm, <20 mm, and >20 mm; as the study had relatively low numbers of sinuses with thickening >10 mm, they grouped the final three grades in their results. Also, as it was determined in this study, the pathology would be defined as anything >2 mm; using a category of <5 mm would not allow separation of normal thickening <2 mm from the pathology—hence, the methodology by Shanbhag et al. (2014) [17] was adopted instead. Measurement of mucosal thickening was in millimetres, using the i-Dixel software measurement tool, taken at the most severe area of thickening perpendicular to the sinus wall as per the methodology described by Janner et al. [18] and adopted by Shanbhag et al. [17]. The thickening was further subcategorised by appearance according to Shanbhag et al. (2014) [17] as normal (no/<2 mm thickening), flat, polypoid, air–fluid level, or complete opacification. The location of the mucosal thickening was recorded as affecting either anterior, posterior, superior, inferior, lateral, and medial walls of the maxillary sinus, as reported by Ahmed et al. [25]; an adaption was made to the classification here to also include circumferential as a category (all walls) taken from the classification by Carmeli et al. [16]. The presence of the ostium was observable, and its patency was also recorded, as per Carmeli et al. [16]. Ostium patency was classified as patent or obstructed, with a blocked ostium at the nasal cavity side considered an obstruction (Figure 1).

#### 2.3.3. Dentition Status

In relation to the status of the patient’s dentition in the posterior maxilla, the following data were recorded:

Whether the patient was partially dentate or edentulous.

The presence of endodontically treated teeth.

The presence of any dental implants and whether sinus floor perforation was seen.

The presence of periodontal disease, as described by Janner et al. (2020) [18], was indicated by marginal bone loss deeper than the midlevel of the respective root or furcation involvement.

The presence of periapical lesions according to the definition by Low et al. [26], where a periapical lesion was recorded if “the width of the radiolucency exceeded at least twice the width of the periodontal ligament space.” The lesion also had to be visible in two image planes on CBCT.

**Figure 2:**
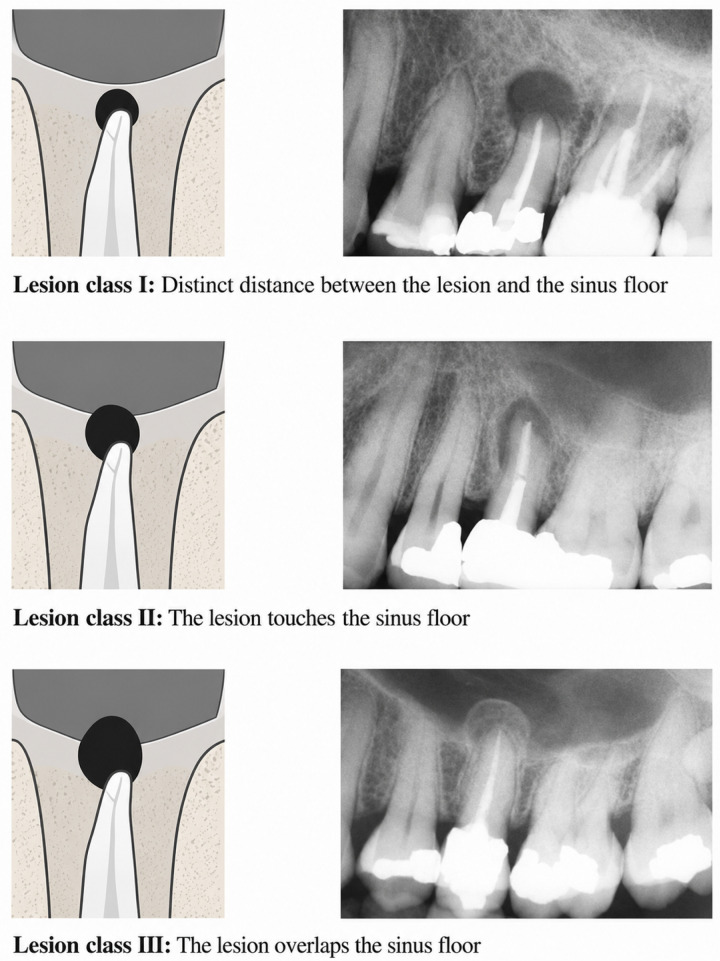
Schematic diagrams and example intraoral periapical radiographs depicting the Oberli classification of apical pathology and its relation to the sinus floor taken from Oberli et al. [27].

The periapical lesions were also classed according to their relation to the sinus floor, as described by Oberli et al. [27] (Figure 2):

Class I: Distinct distance between the lesion and sinus floor;

Class II: Lesion touches the sinus floor;

Class III: Lesion overlaps the sinus floor.

### 2.4. Statistical Analysis

Descriptive statistics were used to summarise demographic characteristics and radiographic findings, including the degree, morphology, and location of mucosal thickening, ostium patency, and associated odontogenic findings. Given the exploratory retrospective design, small sample size, and limited number of obstructed ostia, the study was not designed or powered to support formal inferential statistical analysis. Exploratory Fisher’s Exact testing was included only as a descriptive hypothesis-generating exercise to contextualise observed radiographic patterns and should not be interpreted as evidence of analytical association or predictive relationship. As some patients contributed bilateral sinus observations, the patient was considered the primary unit of analysis. Sinus-level findings were analysed descriptively and were not treated as fully independent observations for inferential interpretation. A sensitivity analysis using alternative mucosal thickening thresholds (>3 mm and >4 mm) was also performed to assess the robustness of the descriptive findings. Although the frequency of mucosal thickening decreased with increasing thresholds, the overall radiographic patterns observed within the cohort remained broadly similar. The anonymised dataset used for the exploratory Fisher’s Exact analysis has been provided as Supplementary Material Table S1.

## 3. Results

### 3.1. Patient Demographics

**Figure 3:**
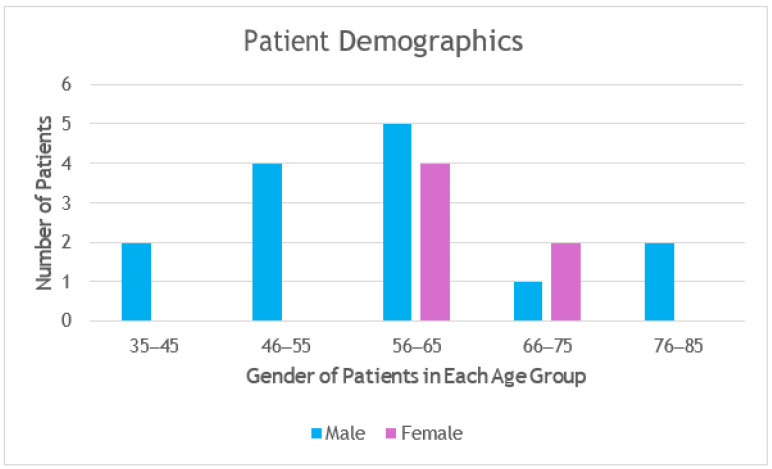
Patient demographics graph displaying the gender breakdown and total number of patients within each age group.

Of the 22 patients whose CBCT scans were assessed, 20 were eligible for inclusion. One was excluded as the sinus surgery they had was to remove a dental implant from the maxillary sinus rather than sinus floor elevation, and the other was excluded as their CBCT could not be located on the system. Only a limited number of patients had bilateral CBCT scans available, which restricted the ability to perform intra-individual comparisons between sinuses. Of the 20 patients included, 14 were males, and six were females, with ages ranging between 37 and 80, and a mean age of 60 (Figure 3). The distribution of participants was skewed towards male patients, with a lower proportion of female individuals. Therefore, any observations related to sex should be considered descriptive only and interpreted with caution.

### 3.2. Degree of Mucosal Thickening

Eighteen of the maxillary sinuses examined presented with mucosal thickening greater than 2 mm at an incidence of 69%. The prevalence of mucosal thickening in this cohort was 69%. This figure represents a centre-specific observation derived from a preselected cohort of patients undergoing assessment for sinus floor elevation, and should not be interpreted as a population-based estimate.

**Figure 4:**
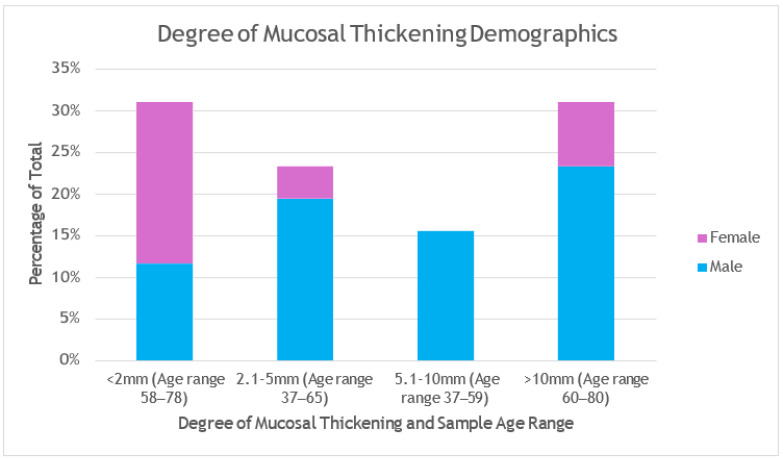
Graph showing the incidence of the varying degrees of mucosal thickening and the demographic composition of each group.

Eight sinuses (31%) were normal with no or <2 mm thickening. Six sinuses (23%) had 2.1–5 mm, four (15%) had 5.1–10 mm, and eight (31%) had mucosal thickening >10 mm. Of the sinuses that had mucosal thickening >2 mm, 15 (83%) sinuses belonged to male patients, and three (17%) were female and were aged between 37 and 80 years old. Of the normal sinuses, three (37.5%) belonged to males, and five (62.5%) were female patients and were aged between 58 and 78 years old. Looking at male and female sinuses separately, 37.5% (3) of female sinuses had thickening >2 mm, whereas 83% (15) of male sinuses had thickening >2 mm. See Figure 4 for the pattern observed between mucosal thickening and patient demographic factors.

### 3.3. Mucosal Thickening Morphology

**Figure 5:**
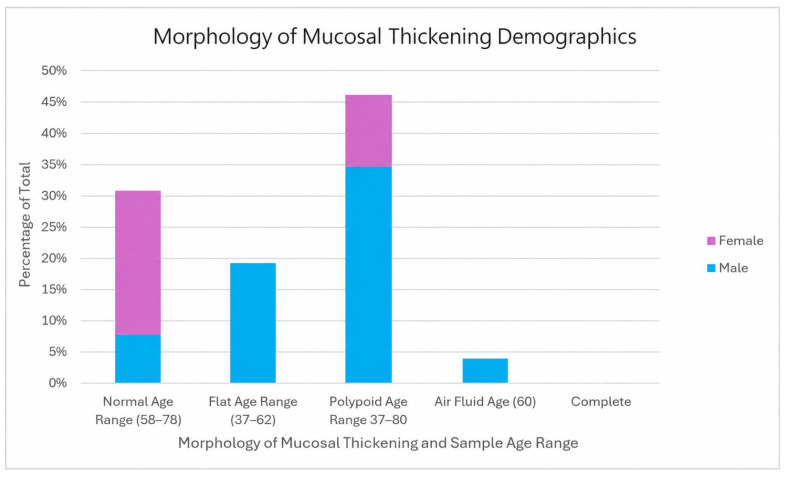
Incidence of varying mucosal thickening morphologies and demographic composition of each group.

**Figure 6:**
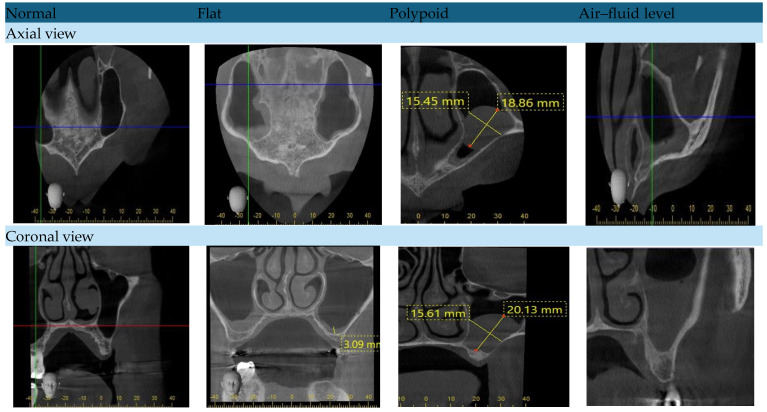
Examples of the varying mucosal thickening morphologies seen in axial and coronal views on CBCT.

Twelve (46%) sinuses had polypoid mucosal thickening; of the patients who had this type of thickening, nine were male, and three were female, aged between 37 and 80 years old. Five (19%) sinuses had flat mucosal thickening; all the patients who had flat mucosal thickening were male and were aged between 37 and 62 years old. One (4%) sinus had an air–fluid level indicative of acute sinusitis. This patient was a 60-year-old male. None (0%) of the sinuses showed complete opacification. See Figure 5 for patterns observed between mucosal thickening and patient demographic factors. Examples of the varying mucosal thickening morphologies seen on the CBCT scans in axial and coronal views can be seen in Figure 6. As none of the patients had complete opacification, there is no example of this.

### 3.4. Mucosal Thickening Location

**Figure 7:**
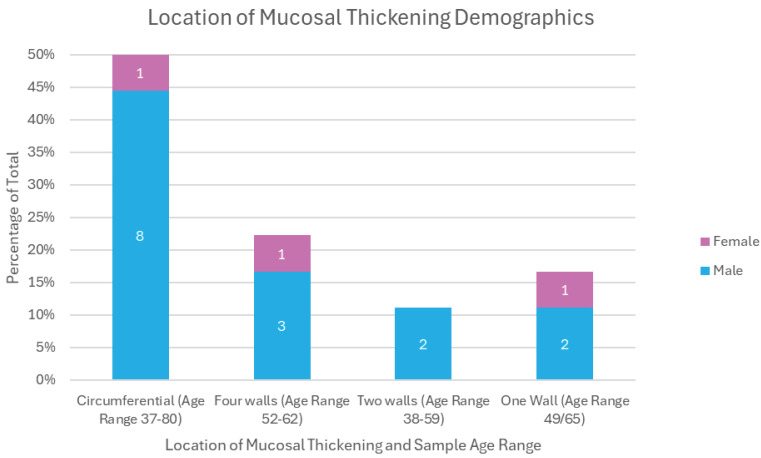
Graph showing the incidence of the varying mucosal thickening morphologies and the demographic composition of each group.

Of the sinuses that had mucosal thickening, nine (50%) had circumferential thickening; of these, eight were male, and one was female, and were aged between 37 and 80 years old. Four (22%) sinuses had mucosal thickening of four walls (anterior, medial, lateral, and inferior walls, but not the posterior); of the patients who had four walls affected, three were male, and one was female, and were aged between 52 and 62. Three (17%) sinuses only had one wall (inferior/medial/anterior) affected. Of these, two patients were male, and one was female, and were aged between 49 and 65 years old. Two (11%) of the sinuses had two (inferior/lateral and posterior/inferior) walls affected; both these patients were male and aged between 38 and 59. See Figure 7 for patterns observed between the location of mucosal thickening and patient demographic factors. The two sinuses that had ostium obstruction had circumferential thickening. Of the nine patients who had circumferential thickening, five did not have a CBCT with a large enough FOV to visualise the ostium.

### 3.5. Membrane Characteristics and Ostium Patency

**Figure 8:**
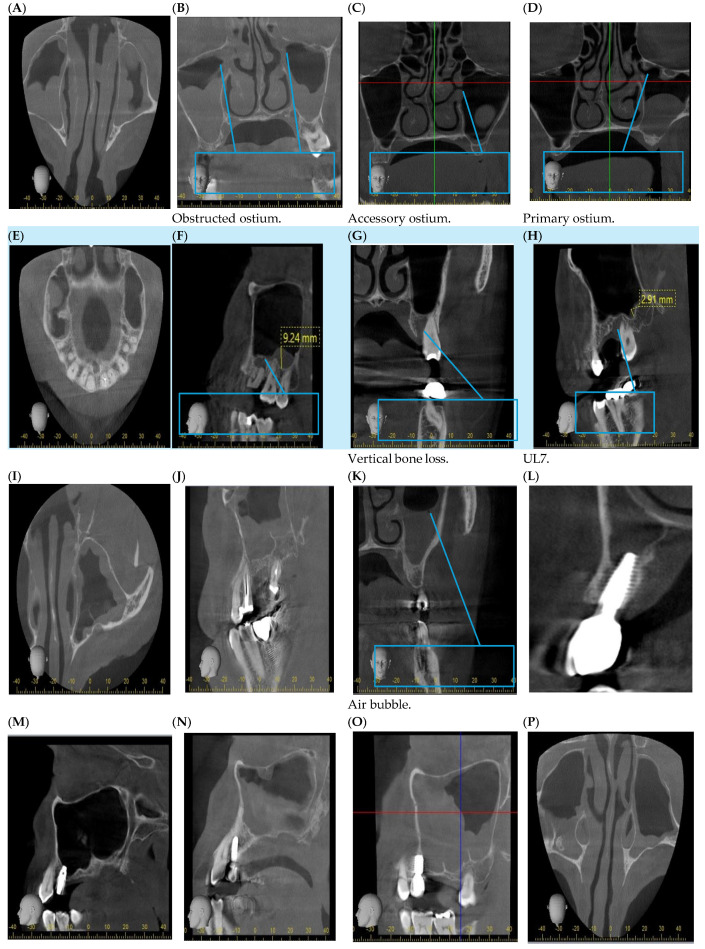
Representative CBCT images of incidental findings in the maxillary sinus. (**A**,**B**) Bilateral polypoid, circumferential mucosal thickening >10 mm with ostium obstruction. (**C**,**D**) Accessory ostium with polypoid thickening >10 mm and patent primary and accessory ostia. (**E**,**F**) Apical pathology (UR6) associated with unilateral polypoid thickening (5–10 mm). (**G**,**H**) Periodontal bone loss (UL7) with flat mucosal thickening (2–5 mm). (**I**,**J**) Periodontal disease and root-filled teeth associated with circumferential polypoid thickening >10 mm. (**K**,**L**) Air–fluid level associated with implant breaching the sinus floor (UL4). (**M**) Implant breaching sinus floor without mucosal thickening. (**N**,**O**) Bilateral implants (UR5, UL4) with circumferential polypoid thickening >10 mm and ostium obstruction. (**P**) Antrolith within the right maxillary sinus. Abbreviations: UR = upper right; UL = upper left.

Of the five sinuses that had flat mucosal thickening, three (60%) had thickening 2.1–5 mm, one (20%) had 5.1–10 mm, and one (20%) had >10 mm. In relation to the location of the flat thickening, three (60%) sinuses had thickening of four walls, one (20%) sinus had one wall, and one (20%) had circumferential thickening. Twelve sinuses had polypoid mucosal thickening. Of these, three (25%) had thickening 2.1–5 mm, three (25%) had 5.1–10 mm, and six (50%) had >10 mm. Of the sinuses with polypoid mucosal thickening, seven (58%) had circumferential thickening, two (17%) had thickening of two walls, two (17%) had thickening of one wall, and one (8%) had four walls affected. One sinus had an air–fluid level, and the thickening here was circumferential and >10 mm. Of the 16 sinuses where the ostium could be visualised, 14 were patent and two (12.5%) were obstructed. See Table 2 for the patterns observed between membrane characteristics and ostium patency. Of the two sinuses that had ostium obstruction, these were the right and left sinuses of a 63-year-old male who presented with mucosal thickening of >10 mm in both sinuses, which was circumferential in location and polypoid in morphology (see Figure 8A,B). The incidence of accessory ostia was 6%, one of the CBCT scans of bilateral sinuses presented with an accessory ostium in the left sinus, this sinus had a polypoid thickening >10 mm affecting four walls (anterior, lateral, medial, and inferior), whilst the right sinus was normal, both the primary and accessory ostia in the left sinus were patent (Figure 8C,D).

Exploratory Fisher’s Exact testing demonstrated no statistically significant association/patterns observed between ostium obstruction and mucosal thickening >10 mm (*p* = 0.083) or polypoid morphology (*p* = 0.175). Circumferential mucosal thickening demonstrated an exploratory association with ostium obstruction (*p* = 0.050). However, these findings should be interpreted with considerable caution due to the very limited number of obstructed ostia and the small sample size.

An exploratory threshold sensitivity assessment was undertaken based on the grouped mucosal thickening categories available within the dataset. As measurements were originally categorised rather than retained as continuous variables, precise recalculation using alternative thresholds could not be performed. However, when considering alternative thresholds of >3 mm or >4 mm, the observed frequency of mucosal thickening within the cohort would remain between 46% and 69%, indicating that the descriptive observation of relatively frequent mucosal thickening within this clinical cohort remained broadly consistent despite threshold variation.

### 3.6. Odontogenic Findings

In assessing the relation between odontogenic causes of unilateral sinusitis, only six patients in the present study had CBCT scans, which encompassed bilateral sinuses. Of the three patients that has unilateral thickening, only one was associated with an odontogenic cause, in which an UR6 had apical pathology. The other three patients who had bilateral sinus scans demonstrated bilateral mucosal thickening. In relation to the other patients who had odontogenic factors observed in their CBCT scans, the scans were only of a single sinus, and as a result, an assessment could not be made as to whether the thickening demonstrated was unilateral. The patient who presented with apical pathology of the UR6 was a 59-year-old male, who, according to the Oberli et al. (2007) [27] classification, was a grade III periapical lesion where it overlapped the sinus floor (Figure 2). This patient had polypoid mucosal thickening between 5.1 and 10 mm affecting the lateral and inferior walls of the right maxillary sinus with a patent ostium, whilst the left maxillary sinus for the same patient was normal. This patient also had evidence of periodontal disease; see Figure 8E,F. Three patients had evidence of periodontal disease as described by Janner et al. (2020) [18] were there was marginal bone loss deeper than the midlevel of the respective root. These patients were all male, aged between 55 and 72 years old. The first of these was the patient with apical pathology of the UR6. Another patient had flat mucosal thickening between 2.1 and 5 mm affecting the inferior wall of the left sinus with a patent ostium and had vertical bone loss in relation to their upper left second molar (UL7); see Figure 8E,F. The final patient had circumferential polypoid thickening of their left sinus >10 mm. This patient had a patent ostium and had two root-filled teeth in the upper left quadrant, which were not associated with any apical pathology; see Figure 8I,J. Further incidental odontogenic findings included three patients who had dental implants in situ—these patients were aged between 58 and 63, where two were male, and one was female. One of these patients had an implant in the UL4 position, which was breaching the sinus floor by 3 mm. An air–fluid level in the left maxillary sinus was seen, which was indicative of acute sinusitis. The mucosal thickening was circumferential and >10 mm; however, the ostium could not be viewed on this CBCT, so patency could not be confirmed see Figure 8K,L. Another patient had an implant in the upper right first premolar (UR4) position, which was also breaching the sinus floor by 3 mm; however, this patient’s sinus was of normal appearance—see Figure 8M. The final patient mentioned earlier and seen in Figure 8A,B had two dental implants in the UR5 and UL4 positions. This patient’s UL4 implant was distant from the sinus floor; the UR5 implant was close to but not breaching the sinus floor—see Figure 8N,O. The final incidental finding was a patient who had an antrolith in situ; see Figure 8P. This was the same patient mentioned earlier in Figure 8A,B,N,O, with bilateral polypoid circumferential mucosal thickening >10 mm with bilateral ostium obstruction.

**Figure 9:**
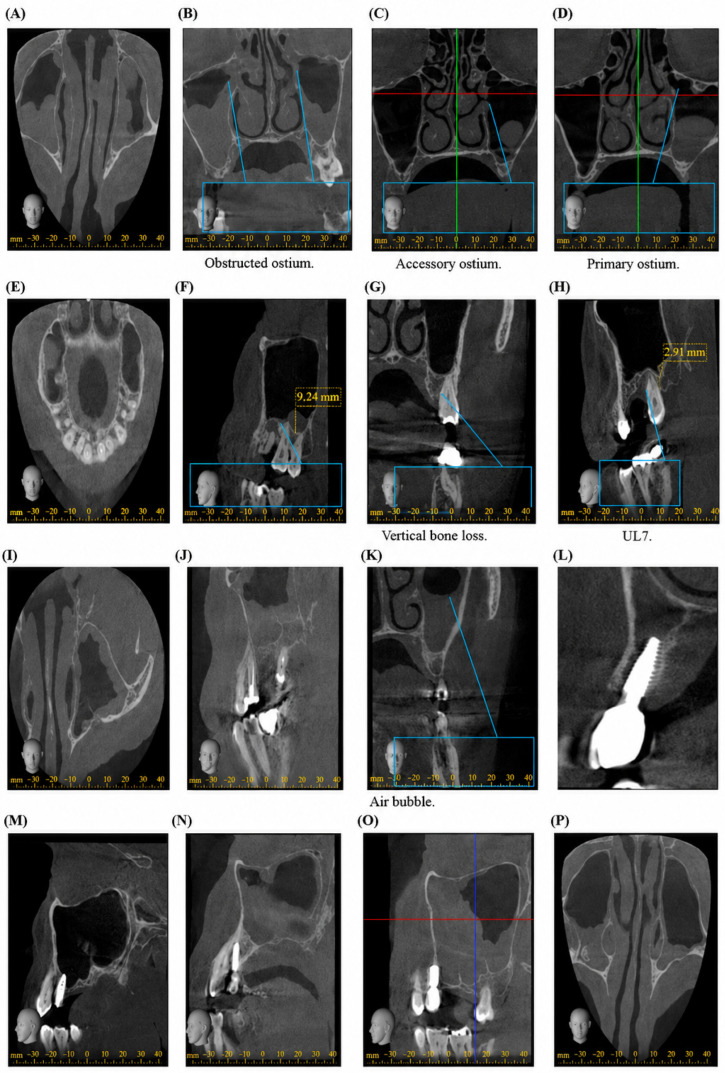
CBCT sections of sinuses that would warrant onward referral based on the framework by Janner et al. (2020) [18]. (**A**). Left maxillary sinus with an air–fluid level occupying >50% sinus; the ostium cannot be seen. (**B**). Polypoid lesion occupying the left maxillary sinus exceeding 50% sinus volume. (**C**). Bilateral sinuses with polypoid thickening occupying >50% sinus volume with bilateral ostium obstruction. (**D**–**F**) One right and two left maxillary sinuses with circumferential thickening and ostium cannot be visualised. (**G**,**H**). Coronal and sagittal CBCT views showing vertical bone loss at UL7 with flat sinus floor thickening of 2–5 mm. (**I**,**J**). Axial and sagittal CBCT views showing periodontal disease and root-filled teeth with circumferential polypoid thickening >10 mm. (**K**,**L**). Coronal and sagittal CBCT views showing an air–fluid level associated with a UL4 implant breaching the sinus floor. (**M**). Sagittal CBCT view showing a UL4 implant breaching the sinus floor without mucosal thickening. (**N**,**O**). Sagittal CBCT views showing implants UR5 and UL4 with bilateral polypoid circumferential thickening >10 mm and bilateral ostium obstruction. (**P**). Axial CBCT view showing an antrolith in the superior anterolateral aspect of the right maxillary sinus.

Referral considerations were descriptively explored in relation to the framework proposed by Janner et al. (2020) [18]; however, formal application of the framework was restricted to cases in which ostium patency could be adequately assessed on CBCT imaging. In cases where the osteomeatal complex could not be reliably visualised due to field-of-view limitations, no definitive classification according to the Janner framework was assigned. These cases were instead described as radiographic findings that may warrant further clinical or ENT assessment based on the extent and morphology of mucosal changes. Seven sinuses (27%) in the present study would have required onward referral to ENT. One required it because there was an air–fluid level >50% with circumferential thickening, and the ostium could not be seen (Figure 9A). Three sinuses required it because opacification exceeded over 50%; in one of these, there were primary and accessory ostia present, both of which were patent (Figure 9B). The other had bilateral ostium obstruction (Figure 9C). Finally, three sinuses because they had circumferential thickening, and the ostium could not be viewed on the CBCT (Figure 9D–F).

## 4. Discussion

This retrospective study examined 26 maxillary sinuses from 20 patients scheduled for sinus floor elevation (SFE) using CBCT imaging. The mean age was 60 years (range: 37–80), with a higher prevalence and greater degree of mucosal thickening in males compared to females, consistent with previous findings [17,27,28]. Pathological mucosal thickening (>2 mm) was present in 69% of sinuses—higher than the 34.9–64.5% range reported in Costa et al. (2018) [8]. Thickening >10 mm was more common in older patients (≥60 years), supporting associations between age and severity of thickening noted by Shanbhag et al. (2014) [17] and Da Silva et al. (2017) [29].

Polypoid morphology was most frequent (46%), followed by flat (19%) and air–fluid levels (4%), with no complete opacification observed. Ostium obstruction was detected in 12.5% of sinuses where the osteomeatal complex was visible, exclusively in cases of circumferential, polypoid thickening >10 mm. These findings align with Carmeli et al. (2011) [16] and Shanbhag et al. (2014) [17], who reported increased obstruction risk with greater thickening and certain morphologies. The absence of complete opacification in this sample reflects the generally low reported prevalence (1–10%), but literature confirms it is strongly associated with ostium obstruction [4,15].

Most CBCT scans in this study were medium field-of-view (FOV), which did not always allow full visualisation of the osteomeatal complex. This may have led to an underestimation of ostium obstruction prevalence. While some authors recommend large FOV imaging to include the osteomeatal complex for drainage assessment [8,15], others highlight increased radiation exposure, adherence to the ALARA principle, and the need for adequate diagnostic expertise [17].

Odontogenic sources of sinus pathology were observed, including periapical lesions, severe periodontal disease, and dental implants breaching the sinus floor. One case involved a Class III periapical lesion in direct contact with the sinus floor and associated with adjacent mucosal thickening, in agreement with Lu et al. (2012) and Maillet et al. (2011) [30,31], who reported a strong link between apical pathology and sinus membrane changes. Periodontal disease was present in three sinuses, all severe, supporting previous studies that associate advanced periodontal involvement with increased mucosal thickening [28,32]. Two patients had implants penetrating the sinus floor; one showed acute sinusitis with an air–fluid level, reflecting literature that penetration >2 mm can predispose to sinus complications [27,31]. One case of an amorphous antrolith was identified in patterns observed with polypoid, circumferential thickening >10 mm and ostium obstruction. Although rare (0.56–3.2% prevalence) [21], antroliths are linked to chronic sinusitis and can require removal when symptomatic. Mucosal thickening is a common radiographic finding and, in isolation, should not be considered a contraindication to sinus floor elevation. However, the clinical relevance depends on specific radiographic characteristics rather than presence alone. A risk-based interpretation is therefore essential when integrating CBCT findings into treatment planning. In a proportion of cases, the field of view did not allow visualisation of the osteomeatal complex, and therefore ostium patency could not be assessed. In these instances, referral considerations were guided by other radiographic features, including the extent and morphology of mucosal thickening and the presence of air–fluid levels. This highlights the importance of adequate field of view selection, as larger FOV CBCT imaging may provide more comprehensive assessment when applying structured referral frameworks such as that proposed by Janner et al.

Overall, this study demonstrates a high prevalence of maxillary sinus pathology in patients assessed for SFE. Within this cohort, ostium obstruction was only observed in cases demonstrating circumferential, polypoid thickening >10 mm; however, due to the very limited number of obstructed cases, this should be interpreted as a descriptive observation rather than evidence of association. The findings highlight the need for comprehensive preoperative CBCT evaluation, consideration of appropriate FOV for drainage pathway assessment, and identification and management of odontogenic pathology prior to SFE to optimise surgical outcomes and reduce postoperative complications.

### 4.1. Referral

The study descriptively explored selected radiographic features discussed within the framework proposed by Janner et al. (2020) [18]. Seven sinuses (27%) in this study met the criteria for onward ENT evaluation before SFE. One case involved a dome-shaped opacification occupying more than 50% of the sinus volume, which could have been aspirated or removed at the time of SFE. However, this sinus also had an accessory ostium, a feature known to disrupt mucociliary clearance [33] and reported in 30% of chronic rhinosinusitis cases [34], warranting ENT referral. Although rounded lesions have a low risk of ostium obstruction (6.1%) (Carmeli et al., 2011), large or symptomatic lesions are generally recommended for removal and ENT assessment [15,16,35,36].

Of the seven referral cases, six had circumferential thickening, one had an air–fluid level, and the remainder were a mix of flat and polypoid forms. Carmeli et al. [16] recommended ENT referral for irregular (>5 mm), circumferential, or complete thickening, as these features increase obstruction risk. Two sinuses with obstruction in this study also met Shanbhag et al.’s (2014) [17] referral criteria for thickened or polypoid mucosa with an obstructed ostium. Because of the retrospective design, it was not possible to assess patients’ sinonasal symptoms, history of rhinosinusitis, or whether ENT referrals were actually made.

### 4.2. Limitations

This study’s small sample size limits the generalisability of findings. All CBCTs were evaluated by a single investigator, introducing potential observer bias. Unlike Shanbhag et al. [17], who calibrated measurements with expert guidance, or Carmeli et al. [16], who used multiple evaluators, this study lacked interobserver calibration. In addition, not all scans included the osteomeatal complex, restricting assessment of the relationship between mucosal thickening and ostium patency. This study should be interpreted within the context of its design as a descriptive, exploratory case series based on a defined clinical cohort. The sample size and distribution of findings reflect real-world clinical practice and were not intended to support inferential or comparative statistical analysis. As some patients contributed bilateral sinuses, the analysis was conducted at the sinus level for descriptive purposes, and potential intra-patient correlation should be considered when interpreting the findings. Future studies with larger cohorts and appropriate statistical modelling are recommended to further explore these observations. This study has several limitations. The relatively small sample size, along with an uneven gender distribution, limits the ability to draw meaningful comparisons between male and female patients. As such, any observed differences are descriptive and should be interpreted with caution. In addition, only a limited number of patients had bilateral CBCT scans, which restricted intra-individual comparisons and reduced the ability to assess unilateral versus bilateral sinus findings. These factors may limit the generalisability of the results.

A low pathological threshold (>2 mm) was used for mucosal thickening, which, while common in the literature [4,8], may risk over-reporting [17]. Other potential influencing factors—such as smoking, asthma, allergies, or recent dental extractions—were not available, meaning some thickening could have been transient or post-extraction related [27]. Periodontal disease classification relied solely on radiographic bone levels [17] without clinical attachment measurements, which some authors view as essential for diagnosis [14,27]. Understanding the prevalence and characteristics of sinus pathology is important when planning sinus floor elevation, as untreated sinus disease may increase surgical complications and compromise implant outcomes. Sinus pathology should be correlated with clinical data when diagnosing rhinosinusitis. It should be noted that, due to the retrospective radiographic design, detailed clinical data (including sinonasal symptoms, ENT history, and recent dental interventions) were not consistently available. As mucosal thickening represents a non-specific radiographic finding, its presence does not necessarily indicate clinically significant sinus disease. Therefore, interpretation of these findings should be made in conjunction with appropriate clinical assessment when informing diagnosis or management decisions. Importantly, only two obstructed ostia were identified within the study cohort, both occurring bilaterally in a single patient. Therefore, inferential statistical findings relating to ostium obstruction are underpowered and should be interpreted as exploratory and hypothesis-generating only.

Although mucosal thickening is frequently identified on CBCT scans, its presence alone does not necessarily contraindicate sinus floor elevation. Several studies have demonstrated that mild to moderate mucosal thickening identified as an incidental radiographic finding may not significantly affect surgical outcomes when the sinus cavity is ventilating and the ostium is patent. However, extensive thickening, particularly when associated with circumferential involvement, ostium obstruction, or odontogenic infection, may increase the risk of postoperative sinus complications. Therefore, appropriate decision-making based on careful radiographic evaluation combined with appropriate clinical assessment is essential when determining whether sinus pathology requires management or referral prior to SFE.

### 4.3. Future Directions

Most research in this area is retrospective. Larger prospective studies are needed to investigate the relationship between preoperative sinonasal symptoms, mucosal thickening, ostium patency, and SFE complication rates. Standardisation in defining pathological thresholds, recording mucosal thickening morphology, and outcome reporting is essential to enable meta-analyses and evidence-based practice. Stronger collaboration between implant dentists and ENT specialists is recommended for patients with sinus pathology that may impair ostium patency. The development of formalised guidelines for classification and referral similar to Janner et al.’s (2020) [18] framework could help clinicians identify which findings warrant ENT involvement, thereby reducing postoperative complications and improving SFE predictability. Future studies with larger sample sizes, a more balanced gender distribution, and a greater number of bilateral CBCT datasets are required to enable more robust subgroup analyses and intra-individual comparisons.

## 5. Conclusions

Within the limitations of this small retrospective descriptive study, more extensive radiographic mucosal changes, including circumferential and polypoid thickening >10 mm, were observed in the small number of cases where ostium obstruction was present. However, due to the exploratory design and extremely limited number of obstructed cases, no analytical conclusions regarding association or prediction can be drawn. These findings reinforce the importance of careful radiographic assessment, appropriate CBCT field-of-view selection, and consideration of odontogenic factors during preoperative planning. Importantly, radiographic mucosal thickening alone does not necessarily indicate clinically significant sinus disease, and findings should always be interpreted in conjunction with clinical assessment. Further, larger, prospective studies with clinical correlation and appropriate statistical analysis are required to better understand the relationship between sinus pathology, ostium patency, and outcomes of sinus floor elevation.

## Figures and Tables

**Table 1:** Showing the varying fields of view of CBCT scans taken.

Field of View (cm)	*n*
8 × 8 × 8.5	11
8 × 8 × 8.6	5
8 × 8 × 4.5	2
4 × 4 × 4	1
8 × 8 × 5	1

**Table 2:** Association between membrane characteristics and ostium patency (based on the CBCT scans of the 16 sinuses where the ostium could be observed).

Membrane Characteristics	Total *n* (%)	Patent *n* (%)	Obstructed *n* (%)
Thickness (mm)			
<2	6 (37.5)	6 (37.5)	0 (0)
2.1–5	3 (18.75)	3 (18.75)	0 (0)
5.1–10	2 (12.5)	2 (12.5)	0 (0)
>10	5 (31.25)	3 (18.75)	2 (12.5)
Morphology			
Normal	6 (37.5)	6 (37.5)	0 (0)
Flat	3 (18.75)	3 (18.75)	0 (0)
Polypoid	7 (43.75)	5 (31.25)	2 (12.5)
Air–fluid level	0 (0)	0 (0)	0 (0)
Complete opacification	0 (0)	0 (0)	0 (0)
Location			
Circumferential	4 (25)	2 (12.5)	2 (12.5)
Four walls	1 (6.25)	1 (6.25)	0 (0)
Two walls	2 (12.5)	2 (12.5)	0 (0)
One wall	3 (18.75)	3 (18.75)	0 (0)
No walls	6 (37.5)	6 (37.5)	0 (0)

## Data Availability

The original contributions presented in this study are included in the article and Supplementary Material. Further inquiries can be directed to the corresponding author.

## Supplementary Materials

The following supporting information can be downloaded at: https://www.mdpi.com/article/10.3390/dj14060340/s1. Table S1. An anonymised dataset used for exploratory Fisher’s Exact analysis of ostium patency and radiographic characteristics in maxillary sinuses assessed by CBCT.
