# Supplementary material for: Descriptive CBCT Findings of Maxillary Sinus Mucosal Changes in Patients Undergoing Sinus Floor Elevation: A Retrospective Observational Study
Source: Dent J (Basel). 2026 Jun 2;14(6):340. doi: 10.3390/dj14060340 (PMC13298208; doi:10.3390/dj14060340)
Supplement: Supplementary file 1 [file dentistry-14-00340-s001.zip › dentistry-4241091-Supplementary Table S1.pdf]

Supplementary Table S1. Anonymised dataset used for exploratory Fisher's Exact analysis of ostium patency and radiographic characteristics in maxillary sinuses assessed by CBCT.

Sinus ID	Sex	Age Range	Thickness Category	Morphology	Location	Ostium Visualised	Ostium Status
S1	Male	58–78	<2 mm	Normal	No walls	Yes	Patent
S2	Female	58–78	<2 mm	Normal	No walls	Yes	Patent
S3	Female	58–78	<2 mm	Normal	No walls	Yes	Patent
S4	Male	58–78	<2 mm	Normal	No walls	Yes	Patent
S5	Female	58–78	<2 mm	Normal	No walls	Yes	Patent
S6	Female	58–78	<2 mm	Normal	No walls	Yes	Patent
S7	Male	37–65	2.1–5 mm	Flat	Four walls	Yes	Patent
S8	Male	37–65	2.1–5 mm	Flat	One wall	Yes	Patent
S9	Male	37–65	2.1–5 mm	Flat	One wall	Yes	Patent
S10	Male	37–59	5.1–10 mm	Polypoid	Two walls	Yes	Patent
S11	Male	37–59	5.1–10 mm	Polypoid	Two walls	Yes	Patent
S12	Male	60–80	>10 mm	Polypoid	Circumferential	Yes	Patent
S13	Female	60–80	>10 mm	Polypoid	Circumferential	Yes	Patent
S14	Male	60–80	>10 mm	Polypoid	One wall	Yes	Patent
S15	Male	60–80	>10 mm	Polypoid	Circumferential	Yes	Obstructed
S16	Male	60–80	>10 mm	Polypoid	Circumferential	Yes	Obstructed

Abbreviations: CBCT = cone-beam computed tomography.

This anonymised supplementary dataset includes only the 16 maxillary sinuses in which the ostium could be adequately visualised and assessed for patency. The dataset was used for the exploratory Fisher's Exact analysis reported in the manuscript.
